# Enhancing Physicians’ Adherence to the 2023 Sudan Malaria Case Management Protocol Using AI as an Intervention Tool

**DOI:** 10.7759/cureus.94184

**Published:** 2025-10-09

**Authors:** Abubakr Muhammed, Samir Ibrahim, Abdulrahman Abbas Yusuf Mohammed, Ahmed Khalid Mohamed Ahmed, Maali Yousif Mustafa Idris, Mohamed Mobark Obed Yousif, Iman Tarig Abdelmohsin Omer, Amer Rababah, Hager Elsir Sherfeldin Mohammed, Samia Ahmed Elbashir Ahmed, Mohammed Osman Ahmed Osman, Zainab Hussein Musa Mohamed, Lugien Ahmed Mohamed Ibrahim, Eiman Yassir Musa Hussain, Hiba Karimeldin Mohamed Ali, Fatima Ahmed Mohamed Mustafa, Suzan Mohammed Eltayeb Eltahir, Musab Elhag Saad Elhag, Abdalmahmoud Asadig Kanan Ahmed

**Affiliations:** 1 Radiology, University of Cape Town, Cape Town, ZAF; 2 Internal Medicine, Mullingar Hospital, Mullingar, IRL; 3 Medicine, University of Gezira, Wad Madani, SDN; 4 Internal Medicine, Al-Managil Teaching Hospital, Managil, SDN; 5 Internal Medicine, Sheikh Khalifa Hospital Fujairah, Fujairah, ARE; 6 Internal Medicine, Bayan University, Khartoum, SDN; 7 Public Health, University of Pennsylvania, Philadelphia, USA; 8 Medicine, Jabal Al-Zaitoon Hospital, Amman, JOR; 9 Internal Medicine, King Abdulaziz Medical City, Riyadh, SAU; 10 Internal Medicine, The National Ribat University, Khartoum, SDN; 11 Dental Public Heath, Sudan Medical Specialization Board, Khartoum, SDN; 12 Molecular Medicine, Institute of Endemic Diseases, Khartoum, SDN; 13 Pulmonary Medicine, Aswan University Hospital, Aswan, EGY; 14 Internal Medicine, Dudley Group NHS Foundation Trust, Stourbridge, GBR; 15 Medicine, Port Sudan Judiciary Clinic, Port Sudan, SDN; 16 Internal Medicine, Sudan Medical Specialization Board, Khartoum, SDN; 17 Medicine, Rayan AlSharaq Polyclinic, Hail, SAU

**Keywords:** artificial intelligence, case management, clinical decision support systems, malaria, physician adherence, treatment protocols

## Abstract

Background and objective: Malaria remains a major health issue in Sudan, and physicians' non-adherence to treatment protocols has negatively impacted patient outcomes. The 2023 Sudan Malaria Case Management Protocol advocated new developments per the national and globally WHO-recommended levels, but there are still gaps in clinical practices. To improve physician compliance and standardize malaria care, decision-support tools can be based on artificial intelligence (AI). This study aims to examine the effect of the introduction of an AI-based clinical decision-support system (CDSS) on the adherence of physicians to the 2023 Sudan Malaria Case Management Protocol at Al-Managil Teaching Hospital (Managil, GZ, SDN).

Methods: A two-cycle clinical audit was conducted between July and September 2025 (baseline audit followed by AI intervention and then re-audit after AI intervention). The current practice and pre-intervention knowledge (n=50 physicians) were evaluated through questionnaires. An AI-CDSS was implemented together with training, incorporating diagnostics, treatment recommendations, and special group advice (pregnancy, neonatal, and G6PD). Compliance rates before and after the intervention were compared, with statistical testing including effect sizes and 95% confidence intervals.

Results: Recognition of the 2023 protocol increased from 38% to 100% (risk difference +62%, 95% CI: 48-76; p<0.001), and awareness of intravenous (IV)/intramuscular (IM) artesunate as first-line treatment rose from 53% to 98% (risk difference +45%, 95% CI: 32-58; p<0.001). Attitudes towards severe malaria improved from 15% to 91% (risk difference +76%, 95% CI: 61-91; p<0.001). The timely initiation of treatment within 24 to 48 hours improved to 100% (p < 0.001). Mean compliance increased from 50.7% (95% CI: 42-59) to 96.8% (95% CI: 92-100, p<0.001). Residual deficiencies persisted in microscopy reporting and G6PD testing.

Conclusion: A combination of an AI-driven CDSS and focused training demonstrated a substantial improvement in physician compliance with the 2023 Sudan Malaria Case Management Protocol, particularly in critical care aspects. However, a possible Hawthorne effect and the absence of clinical outcome data (e.g., morbidity, mortality) should be acknowledged as limitations. Greater scale-up of this approach, while addressing infrastructural and digital literacy challenges, could have a major impact on malaria case management and disease burden in Sudan.

## Introduction

Malaria is one of the major public health conditions in Sudan, where a significant number of the population live at risk of developing the infection. The Federal Ministry of Health of Sudan published the revised 2023 Malaria Case Management Protocol, which is expected to unify and enhance case management of malaria, diagnostics, and follow-up in multiple care facilities, including teaching hospitals [1,2]. Despite the presence of such guidelines, issues with adherence to the malaria treatment protocols are reported, such as ignoring the results of the diagnostic test, inadequate dosing, and failure to use recommended medicine [3,4].

A qualitative study among health workers in Northern Sudan found that the systemic barrier to adherence is marked by anti-malarial and diagnostic supply shortages, inadequate training, lack of supervision, distrust of the diagnostic tool, and pressure to prescribe drugs by the patient himself [3]. These obstacles are combined with workforce constraints and malpractice within the community of specialists; thus, efficient and innovative intervention strategies are acutely needed. The 2023 protocol aligns both national recommendations and revised WHO guidelines on severe malaria management that focus on the use of artesunate in severe malaria and an artemisinin-based combination in uncomplicated cases [2,5,6].

Artificial intelligence (AI) demonstrates great potential in improving adherence to medical practices through clinical decision support, personal reminders, and education for both providers and patients. The potential of AI to enhance adherence to chronic disease and medication usage has previously been demonstrated, showing promise in its application for malaria case management in Sudan [7-10]. The integration of AI-based tools in Al-Managil Teaching Hospital (Managil, GZ, SDN) will help streamline adherence of physicians to the 2023 Malaria Case Management Protocol by a large margin, eventually benefiting the health of patients and contributing to malaria control in Sudan as a whole.

## Materials and methods

Study design and setting

This study was approved by the Institutional Review Board of Al-Managil Teaching Hospital (approval no. 2025/Au/045). All personal and clinical data were anonymized to protect patient privacy and confidentiality. This clinical audit was carried out in Al-Managil Teaching Hospital, Sudan, a tertiary hospital that offers inpatient and outpatient care to a mixed population. The audit revolved around the knowledge and compliance of physicians with the 2023 protocol on malaria case management in Sudan, assessed through the use of an AI-based clinical decision support system (CDSS) as an intervention to modify protocol compliance. The audit was conducted per the process of the classical audit cycle (baseline measure → intervention → re-audit). The project started in May 2025 and was concluded in July 2025, and includes data on the pre- and post-evaluation of the intervention.

Study phases and interventions

First Cycle (Pre-intervention State and Root Cause Analysis)

The baseline assessment was conducted between May 1 and May 15, 2025, via a structured questionnaire. Responses showed low awareness of the existing protocol, slow initiation of treatment, little understanding of severe malaria signs, and improper handling of special patient groups. These missing links in workflow and the lack of decision support tools translated into deficiencies.

Interventions

The quality improvement team collaboratively developed a standard malaria clinical decision-support system with infectious disease specialists, IT workers, and management. This AI-based system mapped blood smear image-based malaria parasite recognition; patient demographics (name, age, gender); clinical information (symptoms, diagnosis, severity classification, allergies, laboratory results); treatment recommendations per the current Sudan Malaria Case Management Protocol; and follow-up and special population needs (pregnancy, G6PD testing). Between May 16 and June 15, 2025, a series of clinical-specific training sessions was conducted with all clinical staff, which comprised an awareness session on how AI-facilitated comprehensive and accurate clinical documentation can enhance patient safety and diagnostic accuracy, and adherence to national guidelines. The audit team actively monitored the AI-CDSS daily through both a review of usage logs and outcomes of the cases. Staff were asked to provide feedback so that challenges with integration could be identified. This promoted user engagement and enforced a high compliance level with the protocol-supported AI prompts, thus emphasizing how AI can assist, normalize, and guide clinical decision-making and documentation during the management of malaria.

Second Cycle

The re-audit (June 16-July 15, 2025) showed major improvements in awareness of the protocol (37.9%→100%), timely treatment initiation (60.3%→100%), and recognition of severe malaria (15%→91.2%). However, gaps persisted in microscopy coverage and G6PD testing before primaquine administration.

Data analysis

Data was collected through standardized, anonymous questionnaires using Google Forms (Google LLC, Mountain View, California, United States). Quantitative analysis compared compliance rates before and after intervention, while qualitative review summarized staff feedback on AI tool usability and barriers. Data reliability was ensured through defined standards and multiple reviewers, though the lack of evaluator blinding introduced some bias. Effectiveness was measured by changes in compliance rates and staff feedback, which indicated improved confidence in adhering to the protocol and using the AI-driven CDSS.

## Results

The results of this study show that physician adherence to the 2023 Sudan Malaria Case Management Protocol improved significantly after the intervention. Recognition of the updated protocol rose from 38% to 100% (p < 0.001), and knowledge of the correct initiation timeframe for treatment increased from 60% to 100% (p < 0.001). Major gains were also seen in the recognition of clinical signs of severe malaria (15% to 91%, p < 0.001) and in identifying intravenous (IV)/intramuscular (IM) artesunate as the first-line treatment (54% to 98%, p < 0.001).

In pregnancy-related malaria, adherence improved markedly: awareness of artemether-lumefantrine (AL) as the first-line treatment increased from 64% to 100% (p < 0.001), quinine plus clindamycin as a second-line option grew from 50% to 94% (p < 0.001), and sulfadoxine-pyrimethamine for intermittent preventive treatment in pregnancy (IPTp) went from 38% to 98% (p < 0.001). Similarly, in neonatal care, correct identification of IV artesunate for congenital malaria nearly doubled (48% to 96%, p < 0.001).

Overall, 14 of the 15 domains showed statistically significant improvements (p < 0.001). The only non-significant change was observed in the already high baseline knowledge of AL as the first-line therapy for uncomplicated *Plasmodium falciparum* malaria (94% vs. 100%, p = 0.079). These findings (Table 1) highlight the strongest gains in critical areas of malaria management, particularly the recognition and treatment of severe malaria, management during pregnancy, and neonatal malaria care. Table 2 features the rate of overall compliance with the 2023 Sudan Malaria Case Management Protocol across the two audit cycles.

**Table 1:** Comparison of physician knowledge and adherence to the 2023 Sudan Malaria Case Management Protocol before and after the introduction of AI-driven CDSS This table summarizes the results of two audit cycles (first cycle: baseline assessment; second cycle: post-intervention) evaluating physician knowledge and adherence to malaria case management standards. Values are presented as frequency (n) and percentage (%) of correct responses (n = 50 physicians per cycle). Improvement is calculated relative to baseline. The p-values were calculated using the chi-square test, and a p-value of <0.05 was considered statistically significant. CDSS: Clinical decision support system; AL: Artemether-lumefantrine; MIP: Malaria in pregnancy; IPTp: Intermittent preventive treatment in pregnancy; IV: Intravenous; IM: Intramuscular

Parameter	First cycle N (%)	Second cycle N (%)	Risk difference (%)	95% CI	χ²	p-value
Use of the 2023 protocol	19 (38%)	50 (100%)	62%	47-77	44.93	<0.001
Knows treatment initiation timeframe (24 to 48 hours)	30 (60%)	50 (100%)	40%	25-55	25.00	<0.001
Identify microscopy/rapid diagnostic test as the gold standard	36 (72%)	50 (100%)	28%	13-43	16.28	<0.001
Knows essential features of a microscopy report	20 (40%)	50 (100%)	60%	45-75	42.86	<0.001
Identify AL as the first-line treatment for *P. falciparum*	47 (94%)	50 (100%)	6%	–9-21	3.09	0.079 (NS)
Identify primaquine for* Plasmodium vivax* radical cure	32 (64%)	50 (100%)	36%	22-50	21.95	<0.001
Knows clinical signs of severe malaria	8 (16%)	45 (90%)	74%	59-89	54.96	<0.001
Knows the lab findings of severe malaria	5 (10%)	35 (70%)	60%	45-75	37.50	<0.001
Identify IV/IM artesunate as the first-line treatment for severe malaria	27 (54%)	49 (98%)	44%	30-58	26.54	<0.001
Identify AL as the first-line treatment for malaria in pregnancy (MIP)	32 (64%)	50 (100%)	36%	22-50	21.95	<0.001
Identify IM artesunate site (thigh)	11 (22%)	46 (92%)	70%	55-85	49.98	<0.001
Identify hypoglycemia as an adverse effect of quinine	40 (80%)	50 (100%)	20%	6-34	11.11	0.001
Identify quinine+clindamycin as the second-line treatment for MIP	25 (50%)	47 (94%)	44%	29-59	24.01	<0.001
Identify sulfadoxine-pyrimethamine for IPTp	19 (38%)	49 (98%)	60%	45-75	41.36	<0.001
Identify IV artesunate for congenital malaria	24 (48%)	48 (96%)	48%	34-62	28.57	<0.001

**Table 2:** Overall compliance with the 2023 Sudan Malaria Case Management Protocol across two audit cycles This table presents overall physician compliance with malaria case management standards during the two audit cycles (n = 50 physicians per cycle). Values are presented as frequency (n) and percentage (%). Improvement is reported as an absolute percentage point gain and a relative percentage increase. McNemar’s test was used to compare paired proportions across cycles, with χ² = 23.0 and p < 0.001, indicating statistically significant improvement. A p-value < 0.05 was considered statistically significant. IM: Intramuscular

Cycle	Mean compliance rate N (%)	Improvement (%)	χ² Value	p-value	Conclusions
First cycle	25 (50.7%)	—	—	—	Low baseline, major gaps in severe malaria recognition, and IM site knowledge
Second cycle	48 (96.8%)	+46.1 points (≈91% relative)	23.0	<0.001	Significant overall improvement, near-universal adherence to 2023 protocol

## Discussion

The current clinical audit in Al-Managil Teaching Hospital indicates that the combination of an AI-based CDSS and specific training of physicians was an effective measure to increase compliance with the 2023 Sudan Malaria Case Management Protocol [2]. The intervention created significant changes in several areas of malaria management, including the awareness of protocols, early initiation of treatment, knowledge of severe malaria symptoms, and care of special groups.

The significant rise in protocol recognition (38% to 100%) points to the efficiency of the AI CDSS in enhancing the awareness of guidelines among care providers [1,2]. This observation is especially meaningful. Knowledge of the standardized protocols is at the core of uniform care delivery. The success of 100% compliance with the critical 24- to 48-hour window treatment regimen underscores the role of AI facilitation in optimizing prompt clinical judgment that is critical in mitigating morbidity and mortality due to malaria [3,5].

The significant increase in the detection of clinical (15% to 91%) and laboratory (10% to 70%) signs and/or symptoms of severe malaria, as well as the identification of intravenous/intramuscular use of artesunate as the first-line treatment, exceeds the capability of the AI system to improve clinical understanding and practice [4,6]. These benefits are medically critical considering that timely and proper treatment of severe malaria leads to a considerable decline in mortality rates of affected patients [5,6].

The improved safety-related knowledge, such as the proper names of the sites for IM injection of artesunate and awareness of hypoglycemia induced by quinine, are examples of AI leading to safer prescription practices [7]. Improvements in malaria management during pregnancy, such as the proper choice of first- and second-line drugs and the use of IPT, reflect the capability of the system to support comprehensive and delicate care for vulnerable populations [2,5].

These findings align with the broader evidence demonstrating that digital clinical decision support tools can substantially improve healthcare quality and guideline adherence in resource-limited settings [8-10]. The results particularly resonate with previous research in Sudan that identified systemic barriers to malaria protocol adherence, including supply shortages, inadequate training, limited supervision, and diagnostic distrust [3]. The AI-facilitated intervention was effective in filling in various gaps in knowledge and workflow identified in prior studies and led to a marked increase in protocol adherence. However, issues such as incomplete microscopy reporting and inadequate G6PD testing prior to primaquine intake highlight the limitations of AI tools. These results indicate that technological interventions cannot be used independently of further training, supervision, and other health system reinforcement [3].

Comparable evidence from other resource-limited settings supports these findings. In Tanzania, the DYNAMIC trial showed that digital algorithms integrated with point-of-care diagnostics and mentorship significantly improved symptom and sign assessment in children compared to standard care [11]. Likewise, the algorithm for the management of childhood illness (ALMANACH) mobile-based CDSS increased the appropriateness of antimalarial and antibiotic prescriptions in rural health facilities [12]. In Uganda, interpretable machine learning models have been applied to predict malaria risk, aiding in earlier diagnosis and resource allocation [13]. These parallels suggest that your intervention’s success is part of a broader global trend where AI and digital tools, when paired with human training and supervision, can bridge persistent knowledge-practice gaps in malaria care.

Beyond protocol adherence, AI technologies are also advancing diagnostic accuracy. Deep learning models trained on thick blood smear images have achieved near-perfect accuracy in distinguishing *P. falciparum* and *P. vivax* from uninfected cells [14]. Thick-drop image-based CDSS systems have demonstrated faster diagnostic turnaround compared with expert microscopists, without sacrificing sensitivity or specificity [15]. Similarly, multi-model deep learning frameworks have reached over 96% accuracy in parasite detection, pointing to the potential for scalable diagnostic solutions in endemic regions [16]. Collectively, these innovations highlight how AI can strengthen both the accuracy and timeliness of malaria care, complementing the protocol-focused improvements observed in this audit.

Despite these strengths, several limitations should be noted. The relatively short follow-up period may not fully capture the sustainability of improvements. A potential Hawthorne effect, whereby participants modify behavior because they are aware of being observed, could have influenced outcomes. The absence of clinical endpoint data, such as morbidity and mortality, limits the ability to directly link improved adherence with patient benefit. Furthermore, this was a single-center audit, reducing generalizability. Future research should therefore include longer-term follow-up, blinded evaluations, multicenter designs, and incorporation of patient outcome measures.

Finally, the scalability of AI-based interventions requires careful consideration. While this audit shows that an AI-driven CDSS can be integrated into local workflows, broader adoption will depend on overcoming infrastructural constraints, ensuring stable digital connectivity, and strengthening digital literacy among healthcare providers. Embedding AI tools into national health information systems or mobile health platforms may facilitate scale-up, but sustained supervision and training will remain essential for success.

## Conclusions

This study demonstrates that AI-powered clinical decision support combined with targeted training improves adherence to the 2023 Sudan Malaria Case Management Protocol in a resource-limited setting. The intervention enhanced areas directly linked to patient safety and outcomes. Scaling up such innovative approaches, while addressing persistent system challenges, could substantially reduce the malaria burden in Sudan and similar contexts.
